# Mitochondria, obesity and aging

**DOI:** 10.18632/aging.100518

**Published:** 2012-12-31

**Authors:** Cecile Vernochet, C. Ronald Kahn

**Affiliations:** Section on Integrative Physiology and Metabolism, Joslin Diabetes Center and Department of Medicine, Harvard Medical School, Boston, MA 02215; USA

**Figure 1:**
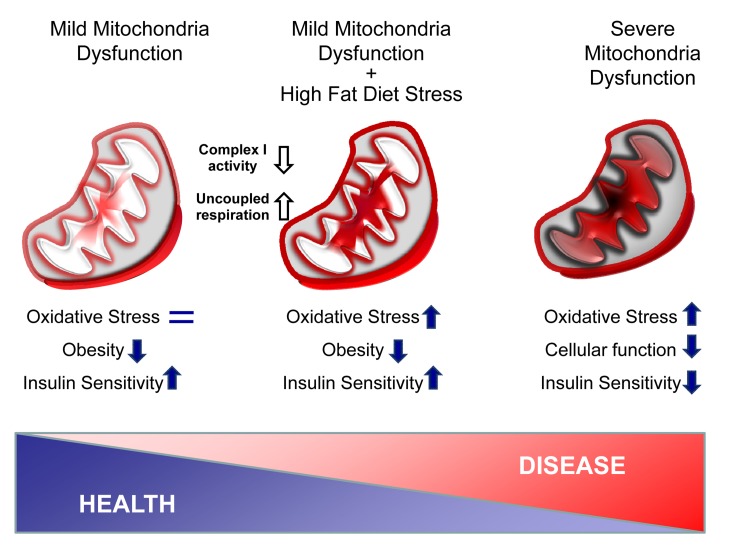
Adipose tissue mitochondria dysfunction protects against obesity and aging-related diseases In TFAM KO adipose tissue, the combination of a decrease in Complex I activity with an increase of uncoupling state creates a mild mitochondria dysfunction without oxidative stress (Left). Upon high fat diet stress, adipose tissue mitochondria are overloaded, but adipose tissue mass remains small and insulin sensitive, despite signs of oxidative stress (Middle). Finally, severe mitochondria dysfunction is known to trigger high level of oxidative stress damage, impairs cellular function and promotes aged-related disorders such as insulin resistance (Right).

Among the many factors contributing to aging, one of the most highly investigated focuses on the theory that there is a gradually decline of mitochondrial function with age leading to progressive tissue damage via oxidative stress (Figure 1, right). Indeed, proper mitochondrial function is required for normal metabolism and health at multiple levels. Mutations in mitochondrial DNA (mtDNA) result in a variety of phenotypes including myopathies, neuropathies, diabetes, signs of premature aging and reduced lifespan [1,2]. Mitochondrial dysfunction in the absence of somatic mutations is also a feature of normal aging and has been observed in species ranging from worms to humans. At the organ level, mitochondrial dysfunction occurs in many age-related diseases, including type 2 diabetes and obesity. In both rodents and humans, obesity and type 2 diabetes are associated reduced expression of mtDNA and reduced levels of proteins involved in oxidative phosphorylation in muscle, liver and adipose tissue [3]. Conversely, caloric restriction, which increases mitochondria biogenesis and maintains mitochondrial function, is associated with increased longevity [2].

Over the past few years, a number of studies have appeared which challenge the mitochondrial theory of aging. Indeed, mutations in genes involved in the electron transport chain that cause mitochondrial dysfunction can sometimes paradoxically lead to improved health and/or enhanced longevity [4]. One example is the situation in mice with conditional knockout of the mitochondrial transcription factor A (TFAM) specifically in fat. These F-TFKO mice exhibit mitochondrial dysfunction with increased energy expenditure, but are protected from age- and diet-induced obesity, insulin resistance and hepatosteatosis, despite increased food intake [5].

Mitochondrial DNA (mtDNA) is maternally inherited with multiple copies in each mitochondria. TFAM plays a critical role in maintenance and expression of mtDNA, and reductions of mtDNA copy number usually correlate with reduction of mitochondria content and function. So, how does a reduction in TFAM in fat have this beneficial effect?

First, despite the reduction in TFAM levels, there is no significant difference in mitochondria number in brown and white adipose tissue between control and F-TFAM KO. Reduction of TFAM, however, does result in decreased mtDNA copy number and altered levels of proteins of the electron transport chain. This in turn results in decreased Complex I activity, greater oxygen consumption and increased uncoupled respiration. As a result, the mitochondrial oxidative capacity of the adipose tissue is increased and outpaces metabolic flux through the TCA cycle, but in mice on a normal chow diet this occurs without indication of oxidative stress or damage (Figure 1, left).

Upon high fat diet, however, the adipose-specific Tfam KO mice develop a build-up of long chain acyl carnitines in both adipose tissue and the circulation. In addition, markers of oxidative stress are observed at the level of DNA and lipids in adipose tissue of F-TFKO mice on high fat diet, indicating overload of the ROS protection system (Figure 1, center). Despite this mitochondria stress, the mice remain lean and insulin sensitive even at 10 months of age. Although no formal aging studies have been conducted in these mice, we also noted that by 18 months of age, an age at which the control mice have started to die, the F-TFKO mice are still thriving, suggesting this knockout may be beneficial to aging mice as well.

White adipose tissue contributes to lipid storage and thermoregulation but is also a critical endocrine organ. In classical endocrine tissues like pancreatic β-cells, mitochondrial dysfunction results in altered insulin secretion and diabetes [3]. In normal lean mice, high levels of adiponectin secretion by adipose tissue are associated with healthy aging and longevity by promoting insulin sensitivity and protecting the heart [6]. Interestingly, although adiponectin mRNA expression is increased in white fat of F-TFKO mice consistent with decreased fat mass, circulating adiponectin levels are reduced by almost 50%. This is because adiponectin peptides undergo multimerization within the ER of adipocytes prior to secretion, and this process can be impaired if mitochondrial activity is reduced and/or uncoupled. While low adiponectin levels in this setting do not appear to promote insulin resistance, cardiac function in the F-TFKO mice has not yet been studied.

Strategies to combat obesity, improve insulin sensitivity and potentially increase longevity include decreasing white adipose tissue mass, increasing brown adipose tissue mass and increasing “browning” of white adipose tissue [7]. In both of the latter situations, there is an overall increase in mitochondrial activity. By contrast, in F-TFKO mice, both white and brown fat mass are reduced and there is no significant “browning” of the white adipose tissue. However, in this setting, increasing mitochondria oxidation in fat has positive metabolic effects that protect mice from obesity, insulin resistance and related pathologies. Whether agents that reduce TFAM level and/or activity or Complex I activity in adipose tissue will reduce of aged related diseases and enhance lifespan remains to be determined.
